# Good Practices and Initiatives for the Control and Elimination of Hepatitis B in the World: A Scoping Review

**DOI:** 10.7759/cureus.59785

**Published:** 2024-05-07

**Authors:** Mohammed A Jalal, Luay M Mohammed, Mustafa Suraifi, Mahshid Namdari, Faris Lami, Taqi Mohammed Jwad Taher, Ayad A Anied, Koorosh Etemad, Manoochehr Karami

**Affiliations:** 1 Department of Epidemiology, School of Public Health & Safety, Shahid Beheshti University of Medical Sciences, Tehran, IRN; 2 Department of Family and Community Medicine, College of Medicine, Baghdad University, Baghdad, IRQ; 3 Department of Family and Community Medicine, Wasit University College of Medicine, Wasit, IRQ; 4 Department of Community Health, Technical Institute of Babylon, Al-Furat Al-Awsat Technical University, Babylon, IRQ

**Keywords:** prevention, screening, vaccination, initiatives, strategies, elimination, control, hepatitis b

## Abstract

Hepatitis B virus (HBV) infection remains a significant global public health challenge, leading to considerable morbidity and mortality. Implementation of effective strategies and novel initiatives is necessary to control and eliminate HBV. To identify the key approaches and actions used worldwide for HBV control and elimination, we conducted a comprehensive scoping review. We searched various sources, including PubMed, Scopus, Web of Science, Google Scholar, the official websites of the World Health Organization (WHO) and the Centers for Disease Control and Prevention (CDC), and relevant articles and reports published in the past decade. Our inclusion criteria focused on studies that reported on strategies for HBV control and elimination, provided evidence of their effectiveness, and assessed their impact on public health outcomes. We included 16 articles in our review, which highlighted a range of strategies, such as universal HBV vaccination, prevention of mother-to-child transmission, mass screening programs, and treatment of chronically infected individuals. These strategies have shown promising results in reducing HBV transmission rate, improving health outcomes, and making progress toward HBV elimination. Moreover, several challenges, including limited access to care, low awareness, stigma, and funding constraints, hinder the effectiveness of elimination programs. The findings underscore the importance of sustained efforts and investment in comprehensive strategies for HBV control and elimination. It is crucial to address barriers to care and enhance public awareness to achieve the goal of eliminating HBV as a public health threat by 2030.

## Introduction and background

Hepatitis B virus (HBV) infection remains a significant global public health concern, affecting an estimated 257 million individuals worldwide with chronic HBV infection and causing approximately 887,000 deaths annually due to complications such as cirrhosis and hepatocellular carcinoma (HCC) [1,2]. Despite the availability of effective preventive measures, such as vaccination and advancements in treatment options, the burden of HBV-related morbidity and mortality persists, particularly in low- and middle-income countries [3]. Controlling and eliminating hepatitis B requires comprehensive approaches that encompass primary prevention, screening, diagnosis, treatment, and public health interventions. Over the past few decades, numerous strategies and initiatives have been implemented globally to combat HBV transmission and reduce its associated burden. These initiatives include universal vaccination programs, mass screening campaigns, health promotion activities, and policy interventions aimed at improving access to care and treatment services [4,5]. However, despite the existence of these interventions, challenges persist in achieving the ambitious goal of hepatitis B elimination by 2030, as outlined in the World Health Organization's Global Health Sector Strategy on Viral Hepatitis (2016-2021). These challenges include barriers to healthcare access, insufficient funding, disparities in vaccination coverage, inadequate surveillance systems, and limited awareness about HBV among the general population and healthcare providers [6,4].

To address these challenges and accelerate progress toward HBV elimination, it is crucial to identify and evaluate the effectiveness of existing strategies and initiatives implemented worldwide [7]. A scoping review offers a comprehensive approach to mapping the breadth of available literature on this topic, synthesizing evidence from various sources to inform future research directions, policy development, and public health interventions [8]. This scoping review aimed to comprehensively examine the strategies and initiatives implemented globally for the control and elimination of hepatitis B. We systematically analyzed the range of interventions employed to prevent, diagnose, and treat hepatitis B. Furthermore, we assessed the existing evidence regarding the effectiveness of these interventions in reducing HBV transmission, improving health outcomes, and advancing progress toward HBV elimination. Additionally, we explored the implications of these findings for policy development, resource allocation, and future research priorities in the field of hepatitis B control and elimination.

## Review

Methodology

Search Strategy

A comprehensive literature search was conducted across multiple electronic databases, including PubMed, Scopus, Web of Science, and Google Scholar. Additionally, reports, guidelines, and publications from official websites of relevant organizations such as the World Health Organization (WHO) and the Centers for Disease Control and Prevention (CDC) were also searched. The search strategy utilized a combination of relevant keywords and Medical Subject Heading (MeSH) terms related to hepatitis B and public health interventions. The search terms were carefully selected to target articles focusing on strategies and initiatives for the control and elimination of hepatitis B. Key concepts included hepatitis B prevention and control, vaccination, mass screening, health promotion, program evaluation, and evidence-based practice. To ensure the search strategy's comprehensiveness and relevance, experienced librarians were consulted in its development.

Eligibility Criteria

The following criteria were considered for inclusion of the primary study and relevant evidence: (i) studies that report on global strategies and initiatives aimed at controlling and eliminating HBV; (ii) the study had to provide evidence demonstrating the effectiveness of interventions related to the control and elimination of HBV; (iii) the focus of the study had to be on public health outcomes, such as incidence rates, prevalence, vaccination coverage, and disease burden. Lastly, the publication had to be within the last 15 years to ensure that it is current and relevant to present-day practices and initiatives.

Studies were excluded if they met any of the following criteria. Firstly, if the study solely focused on hepatitis C or other unrelated infectious diseases, it was not considered for inclusion. Secondly, studies that were not available in the English language were excluded. Thirdly, studies with a narrow regional focus that did not provide broader insights into global strategies for HBV control and elimination were also excluded. Finally, the gray literature, such as conference abstracts and non-peer-reviewed materials, would only be considered if substantial evidence of effective strategies or initiatives was provided.

Study Selection

Two independent groups of reviewers screened the titles and abstracts of the identified articles according to the inclusion and exclusion criteria. Any discrepancies or disagreements that arose were resolved through discussion and consensus. The full texts of articles that were deemed potentially relevant were subsequently assessed for eligibility based on the predefined criteria.

Data Extraction

Data extraction was independently performed by two reviewers using a standardized data extraction form. The extracted data included study characteristics (author, year of publication), study design, population characteristics, intervention details, outcomes measured, and key findings related to intervention effectiveness.

Data Synthesis

The extracted data were narratively synthesized, categorizing identified strategies and initiatives based on their objectives and target populations. A summary of the evidence supporting the effectiveness of these interventions in reducing HBV transmission, improving health outcomes, and advancing progress toward HBV elimination was provided.

PRISMA Flow Diagram

**Figure 1:**
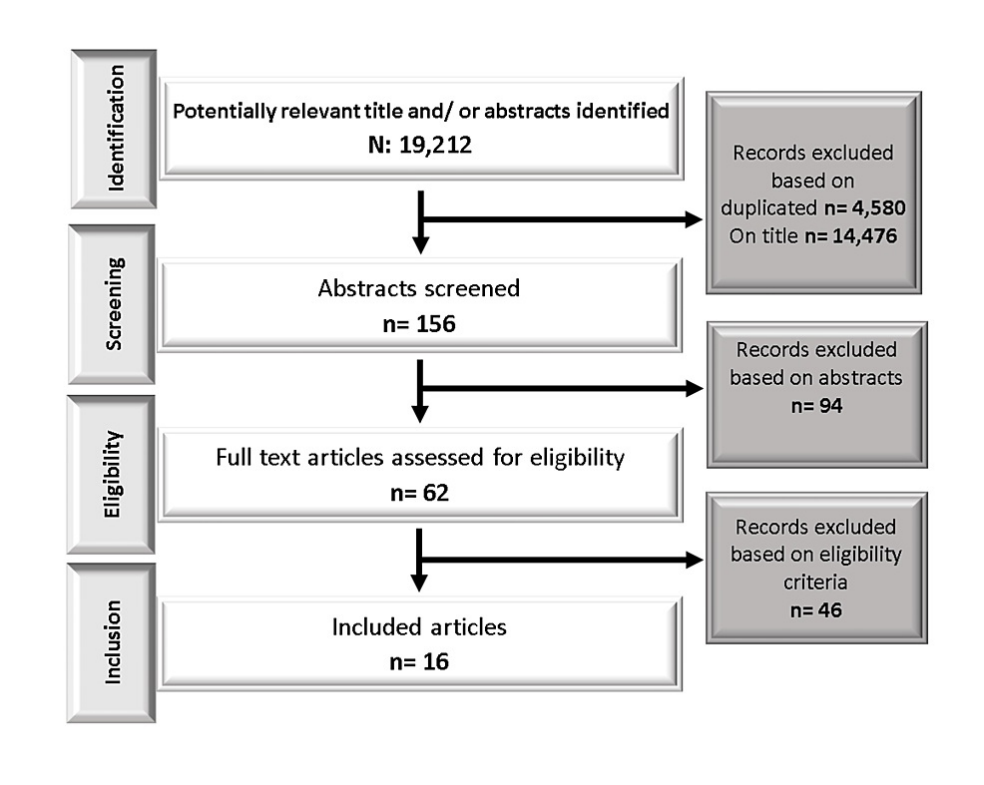
PRISMA flow diagram of retrieved articles included in the scoping review. PRISMA: Preferred Reporting Items for Systematic Reviews and Meta-Analyses.

The Preferred Reporting Items for Systematic Reviews and Meta-Analyses (PRISMA) flow diagram provides a comprehensive overview of the screening and selection process employed in this systematic review. Initially, a total of 19,212 titles and/or abstracts were identified through database searches. After removing duplicates, 14,632 unique records remained for further assessment. The abstracts of these records were screened, resulting in 156 potentially relevant articles for full-text assessment. Following a thorough examination of the full-text articles, 46 records were excluded based on predefined eligibility criteria. Ultimately, 16 articles were deemed eligible and included in the scoping review [4,9-23]. The PRISMA flow diagram showcases a rigorous and transparent process, highlighting the systematic approach taken to identify and select relevant studies for this review (Figure 1).

Results

In the results section of the scoping review, we present a comprehensive summary of the literature on strategies and initiatives for the global control and elimination of HBV. By conducting a rigorous search process and applying specific inclusion criteria, we identified 16 relevant studies that address different aspects of HBV control and elimination efforts. The synthesis of these studies revealed a diverse range of strategies and interventions implemented worldwide, including universal vaccination programs, mass screening initiatives, efforts to prevent mother-to-child transmission, and public awareness campaigns. While progress has been made in certain regions, challenges such as limited access to care, funding constraints, and persistent stigma continue to impede HBV elimination. The findings underscore the ongoing need for research, collaboration, and policy action to address these challenges and advance progress toward the overarching goal of HBV elimination.

The included studies provided valuable insights into various strategies, challenges, and progress related to the control and elimination of HBV on a global scale. Table 1 presents a descriptive overview of the included studies, summarizing key information such as the author, year of publication, title, research site, and journal published from each study.

**Table 1:** Descriptive table of included studies. HBV, hepatitis B virus; HCV, hepatitis C virus; BMC, BioMed Central.

No.	Author & year	Title	Study design	Place of study	Journal published	Sources of access
1	Nawarat Posuwan (2020) [17]	Towards the elimination of viral hepatitis in Thailand by the year 2030	Review	Thailand	Journal of Viral Hepatitis	Scopus, PubMed, Google Scholar
2	Stephanie Popping (2019) [23]	The global campaign to eliminate HBV and HCV infection: International Viral Hepatitis Elimination Meeting and core indicators for development towards the 2030 elimination goals	Review	Global	BMC Infectious Diseases	Scopus, PubMed, Google Scholar
3	Sophia E. Schröeder (2019) [22]	Innovative strategies for the elimination of viral hepatitis at a national level: A country case series	A country case series	Various countries (8 countries)	Journal of Hepatology	Scopus, PubMed, Google Scholar
4	Shevanthi Nayagam (2019) [21]	Strategies for global elimination of chronic HBV infection: 2019 update	Review	Global	Journal of Hepatology	Scopus, PubMed, Google Scholar
5	Shevanthi Nayagam (2016) [4]	Requirements for global elimination of hepatitis B: a modelling study	Modeling study	Global	The Lancet Infectious Diseases	Scopus, PubMed, Google Scholar
6	Shanley Smith (2019) [20]	Global progress on the elimination of viral hepatitis as a major public health threat: An analysis of WHO Member State responses 2017	Analysis	Various countries	Global Health Action	Scopus, PubMed, Google Scholar, WHO
7	Senthilkumar Ramasamy (2024) [19]	Eliminating viral hepatitis from India and Southeast Asia by 2030: challenges and ways forward	Review	India, Southeast Asia	Journal of Clinical and Experimental Hepatology	Scopus, PubMed, Google Scholar
8	Salah T. Al Awaidy (2020) [18]	Moving towards hepatitis B elimination in Gulf Health Council states: From commitment to action	Review	Gulf Health Council states	Public Health Panorama	Scopus, PubMed, Google Scholar, WHO
9	Jie Tang (2021) [16]	Elimination of hepatitis B virus infection in children: experience and challenge in China	Review	China	BMC Public Health	Scopus, PubMed, Google Scholar
10	Jessica Howell (2023) [15]	Pathway to global elimination of hepatitis B: HBV cure is just the first step	Review	Global	The Lancet Gastroenterology & Hepatology	Scopus, PubMed, Google Scholar
11	Jessica Howell (2021) [14]	A global investment framework for the elimination of hepatitis B	Review	Global	BMC Medicine	Scopus, PubMed, Google Scholar
12	Fang-Ting Lu (2020) [13]	Elimination of mother-to-infant transmission of hepatitis B virus: 35 years of experience	Review	Taiwan	Liver International	Scopus, PubMed, Google Scholar
13	Ding-Shinn Chen (2010) [12]	Toward elimination and eradication of hepatitis B	Review	Global	Journal of Gastroenterology and Hepatology	Scopus, PubMed, Google Scholar
14	Chun-Jen Liu (2020) [11]	Elimination of hepatitis B in highly endemic settings: lessons learned in Taiwan and challenges ahead	Review	Taiwan	Journal of Infectious Diseases and Therapy	Scopus, PubMed, Google Scholar
15	Celia Haering (2021) [10]	Hepatitis B virus elimination status and strategies in circumpolar countries, 2020	Review	Circumpolar countries	Journal of Viral Hepatitis	Scopus, PubMed, Google Scholar, WHO
16	Belaynew W. Taye (2022) [9]	Targeted antiviral treatment of hepatitis B virus in culturally and linguistically diverse populations to achieve elimination targets in Australia	Review	Australia	Frontiers in Pharmacology	Scopus, PubMed, Google Scholar

Table 2 provides a concise summary of the strategies discussed in each article, encompassing universal HBV birth-dose vaccination, prevention of mother-to-child transmission, mass screening programs, diagnosis of chronic HBV (CHB), blood supply screening, and health education. It serves as a convenient reference for comprehending the strategies implemented and investigated in the reviewed studies. Table 2 offers a comprehensive overview of the strategies identified for HBV elimination as presented in the included articles.

**Table 2:** Informative strategies for HBV elimination in articles. (✓) indicates the presence of the respective strategy in the article. HBV, hepatitis B virus; CHB, chronic hepatitis B.

No.	Author & year	Strategies for HBV elimination							
		Universal HBV birth-dose vaccine	Prevention of mother-to-child transmission	Universal HBV of high-risk groups vaccine	Mass screening program	CHB diagnosis	Blood supply screening	Treating the chronically infected	(Health education) General public awareness
1	Nawarat Posuwan (2020) [17]	✓	✓		✓		✓	✓	
2	Stephanie Popping (2019) [23]	✓			✓	✓		✓	
3	Sophia E. Schröeder (2019) [22]	✓	✓			✓	✓	✓	
4	Shevanthi Nayagam (2019) [21]	✓	✓		✓			✓	
5	Shevanthi Nayagam (2016) [4]	✓	✓		✓			✓	
6	Shanley Smith (2019) [20]					✓		✓	
7	Senthilkumar Ramasamy (2024) [19]	✓		✓		✓		✓	✓
8	Salah T. Al Awaidy (2020) [18]	✓		✓	✓		✓	✓	
9	Jie Tang (2021) [16]	✓	✓		✓			✓	
10	Jessica Howell (2023) [15]	✓		✓	✓			✓	
11	Jessica Howell (2021) [14]	✓		✓	✓			✓	✓
12	Fang-Ting Lu (2020) [13]	✓	✓		✓			✓	
13	Ding-Shinn Chen (2010) [12]								
14	Chun-Jen Liu (2020) [11]	✓					✓		
15	Celia Haering (2021) [10]	✓	✓		✓	✓		✓	
16	Belaynew W. Taye (2022) [9]				✓			✓	

The authors of the studies emphasize a range of strategies and initiatives for eliminating viral hepatitis, such as universal HBV infant vaccination, prevention of mother-to-child transmission, scaling up interventions, improving diagnosis and treatment, aligning efforts with broader health system goals, and enhancing accessibility and awareness. These strategies have shown progress in hepatitis elimination, including the prevention of new infections through vaccination and reducing transmission in specific regions. However, there are challenges to overcome, including issues related to linkage to care, stigma, funding limitations, and lack of data. The authors emphasize the importance of political commitment, collaboration, sustainable funding, capacity building, and strategic information. To achieve viral hepatitis elimination, a comprehensive approach is required, involving improved healthcare systems, increased awareness, and targeted efforts for high-risk populations. International support and financial investment are particularly crucial for low- and middle-income countries to accelerate progress and meet elimination targets. For more detailed information, please refer to Table 3, which provides a comprehensive summary of the strategies identified in each article, their impact on public health outcomes, and their contributions to the elimination of viral hepatitis.

**Table 3:** Strategies, impact, and contributions toward viral hepatitis elimination. HBV, hepatitis B virus; CHB, chronic hepatitis B; PMTCT, prevention of mother-to-child transmission; GHC, Gulf Health Council; GAVI, Global Alliance on Vaccines and Immunization; HBIG, hepatitis B immune globulin; HBsAg, hepatitis B surface antigen; WHO, World Health Organization; CALD, chronic active liver disease; HCC, hepatocellular carcinoma.

No.	Author & year	Strategies/initiatives	Impact on public health outcomes	Contributions/practical implications
1	Nawarat Posuwan (2020) [17]	Universal HBV infant vaccination, prevention of mother-to-child transmission	Improved socioeconomic status has reduced hepatitis incidence in Thailand	A pilot plan has been proposed in Phetchabun province, Thailand, to eliminate hepatitis B by 2030. The plan focuses on increasing diagnosis rates through widespread HBV testing in high-prevalence areas. Local healthcare personnel conducted affordable HBsAg rapid strip tests at primary health centers, with positive results confirmed at higher-level hospitals. Active HBV cases received priority treatment using antiviral drugs. The plan's effectiveness and cost-effectiveness have been evaluated to guide future public health policies. The ultimate goal is to expand this model nationwide and achieve hepatitis B elimination by 2030.
2	Stephanie Popping (2019) [23]	Mass screening programs, CHB diagnosis, blood supply screening, health education	Challenges to elimination programs include linkage to care, awareness, stigma, and lack of funding	The findings from previous and current initiatives underscored the critical elements necessary for effective elimination programs, which encompass action plans, capacity building, integrated services, strategic data collection, and progress monitoring.
3	Sophia E. Schröeder (2019) [22]	Universal HBV birth-dose vaccine, prevention of mother-to-child transmission, universal HBV of high-risk groups vaccine, mass screening programs, CHB diagnosis, blood supply screening, treating the chronically infected, general public awareness	Major gains in tackling viral hepatitis are possible across different settings	The technological advancements and growing awareness of its risks have made elimination feasible and gained significant political and societal support. Investing in viral hepatitis elimination not only offers health benefits but also aligns with sustainable development goals. Sustaining political momentum is essential, and more countries must take action to achieve global elimination. Successful approaches from diverse income countries provide practical examples for reaching the WHO's 2030 viral hepatitis elimination targets.
4	Shevanthi Nayagam (2019) [21]	Universal HBV infant vaccination, prevention of mother-to-child transmission, mass screening programs, CHB diagnosis, treating the chronically infected	Urgent focus is needed on HBV PMTCT intervention scale-up in Sub-Saharan Africa	To achieve HBV elimination targets, urgent attention is needed for scaling up HBV PMTCT interventions and expanding population-level testing and treatment worldwide. Promising solutions include innovative outreach strategies, novel biomarkers, and the possibility of a functional cure. However, careful financing, policy planning, capacity building, and strong surveillance systems should not be overlooked and must be integral to the elimination efforts.
5	Shevanthi Nayagam (2016) [4]	Universal HBV birth-dose vaccine, prevention of mother-to-child transmission, universal HBV of high-risk groups vaccine, mass screening programs, CHB diagnosis, blood supply screening, treating the chronically infected, general public awareness	Vaccination has prevented 210 million new chronic infections by 2015	The infant vaccination has had significant benefits in addressing HBV, but a new strategy is needed to achieve HBV elimination. This includes expanding prevention interventions for newborns, improving diagnosis and treatment for those with chronic HBV, and expanding infant vaccination programs. These efforts should align with broader health system goals, including addressing chronic diseases and reducing healthcare costs. Innovation in healthcare can support this strategy, and there is a need to replicate the successes of the HIV response in global public health.
6	Shanley Smith (2019) [20]	Mass screening programs, CHB diagnosis, treating the chronically infected	Most people with hepatitis B and C live in countries with national strategies	Progress has been made in national hepatitis planning, but financing remains a challenge. Testing and treatment efforts are still in their early stages in many countries, and there is a lack of strategic information to guide elimination plans. To address these issues, governments should collaborate with civil society and receive support from WHO and other stakeholders. It is important to estimate resource needs, increase investment through innovative financing, strengthen the case for testing and treatment, and enhance strategic information systems. The WHO's Global Reporting System for Hepatitis will continue to monitor progress and trends in the future.
7	Senthilkumar Ramasamy (2024) [19]	Universal HBV infant vaccination, universal HBV of high-risk groups vaccine, mass screening programs, CHB diagnosis, blood supply screening, treating the chronically infected, general public awareness	Services need to be more accessible and holistic for hepatitis eradication	To improve accessibility, increase awareness, and implement a comprehensive approach that encompasses community education, early detection, standardized protocols, infrastructure strengthening, staff training, program integration, and a web-based information system, significant strides can be made in the eradication of hepatitis. This collective effort ensures improved access and establishes a solid foundation for effective hepatitis control and prevention.
8	Salah T. Al Awaidy (2020) [18]	Universal HBV infant vaccination, universal HBV of high-risk groups vaccine, mass screening programs, CHB diagnosis, blood supply screening, treating the chronically infected, general public awareness	GHC states have made progress toward hepatitis B elimination	To decrease HBV-related morbidity and mortality, it is essential to implement interventions. It is crucial for states to adhere to WHO guidelines, develop national strategies, target high-risk populations, enhance surveillance, and improve treatment. Taking prompt and collaborative action is necessary to eliminate the risk posed by hepatitis B.
9	Jie Tang (2021) [16]	Universal HBV infant vaccination, prevention of mother-to-child transmission, universal HBV of high-risk groups vaccine, mass screening programs, CHB diagnosis, treating the chronically infected, general public awareness	Substantial progress has been achieved in eliminating chronic HBV infection in children	China has made significant progress in eliminating chronic HBV infection in children and young adults through universal infant vaccination and free HBIG administration to infants of HBsAg-positive mothers. However, efforts must continue to improve coverage of HBIG and hepatitis B vaccine in newborns and administer oral anti-HBV agents to HBsAg-positive mothers with high viral loads.
10	Jessica Howell (2023) [15]	Universal HBV infant vaccination, universal HBV of high-risk groups vaccine, mass screening programs, CHB diagnosis, blood supply screening, treating the chronically infected, general public awareness	HBV cure is just one part of the solution for HBV elimination	The path to HBV elimination is becoming clearer with promising cure research. However, achieving global elimination requires more than just a cure. We need to optimize current tools, improve the HBV care system, and continue using existing vaccines and treatments to reach mortality targets by 2030. It is important to proactively prepare for the cure while working towards elimination.
11	Jessica Howell (2021) [14]	Universal HBV infant vaccination, universal HBV of high-risk groups vaccine, mass screening programs, CHB diagnosis, blood supply screening, treating the chronically infected, general public awareness	Hepatitis B elimination is achievable with greater commitment from various stakeholders	Eliminating hepatitis B requires commitment from governments, international institutions, civil society, and donors. Countries that are not currently funding elimination efforts need to address affordability and competing priorities. The investment framework suggests raising awareness, reducing stigma, expanding vaccination coverage, improving access to diagnostics and treatment, strengthening health systems, and investing in research for a cure. Financial support from international agencies and donors is crucial for low- and middle-income countries to achieve their elimination goals.
12	Fang-Ting Lu (2020) [13]	Universal HBV infant vaccination, prevention of mother-to-child transmission, universal HBV of high-risk groups vaccine, mass screening programs, CHB diagnosis, blood supply screening, treating the chronically infected, general public awareness	Successful prevention of HBV transmission in Taiwan suggests the possible elimination of HBV worldwide in the future	To accomplish the worldwide elimination of HBV, the implementation of a comprehensive public health program and the establishment of a strong medical care system are essential. Taiwan has achieved noteworthy advancements in this area, including high rates of vaccination coverage, extensive screening of pregnant women, timely administration of antiviral therapy, and a significant decrease in HBV infection rates and related complications. Nevertheless, challenges remain in preventing transmission from highly infectious mothers. It is of utmost importance to prioritize the use of prophylactic treatment and ensure consistent care for pediatric patients with chronic HBV.
13	Ding-Shinn Chen (2010) [12]	Universal HBV birth-dose vaccine, prevention of mother-to-child transmission, universal HBV of high-risk groups vaccine, mass screening programs, CHB diagnosis, blood supply screening, treating the chronically infected, general public awareness	Existing means can prevent and treat HBV infection effectively	The challenges in expanding hepatitis B mass vaccination include improving infrastructure, strengthening education, addressing economic barriers, and securing ongoing support. The Global Alliance on Vaccines and Immunization (GAVI) has played a significant role in supporting vaccination efforts. Combining universal vaccination with effective treatment and interrupting transmission routes offers the potential for HBV elimination. Long-term commitment and sustained efforts are necessary, even if complete eradication is not achieved. Nonetheless, these efforts will significantly reduce HBV infection rates and associated disease burdens.
14	Chun-Jen Liu (2020) [11]	Universal HBV infant vaccination, prevention of mother-to-child transmission, universal HBV of high-risk groups vaccine, mass screening programs, CHB diagnosis, blood supply screening, treating the chronically infected, general public awareness	Vaccination and antiviral reimbursement programs effectively control HBV infections	Reviewed Taiwan's progress toward eliminating HBV through vaccination and antiviral programs. To achieve HBV elimination by 2030, several challenges must be addressed. These include minimizing the risk of mother-to-infant transmission, developing curative HBV treatments, ensuring global accessibility to cost-effective therapies, establishing effective agents for HCC prevention, and implementing lifestyle modifications for concurrent metabolic liver diseases. Vaccination and reimbursement programs have been effective, but further efforts are needed to overcome these remaining hurdles.
15	Celia Haering (2021) [10]	Universal HBV infant vaccination, prevention of mother-to-child transmission, universal HBV of high-risk groups vaccine, mass screening programs, CHB diagnosis, blood supply screening, treating the chronically infected, general public awareness	Data on WHO targets and progress is lacking in circumpolar countries	Coordinated efforts and resource investment are needed among the WHO, national governments, regional bodies, and civil society organizations to eliminate HBV infection. National elimination planning is crucial for identifying opportunities within healthcare systems to reduce HBV-related illness and mortality. Circumpolar countries have had mixed success in meeting targets and developing elimination plans, highlighting the importance of establishing measurable targets and improving data through increased screening.
16	Belaynew W. Taye (2022) [9]	Universal HBV infant vaccination, universal HBV of high-risk groups vaccine, mass screening programs, CHB diagnosis, blood supply screening, treating the chronically infected, general public awareness	Targeted antiviral treatment for CALD populations reduces HBV-related complications and mortality	Discussed targeted antiviral treatment for culturally and linguistically diverse populations in Australia to achieve elimination targets.

Discussion

The discussion section critically analyzes the findings of the scoping review, situates them within the existing literature, and identifies their implications for practice, policy, and future research.

Key Strategies and Initiatives for HBV Control and Elimination

This review examines various strategies and initiatives aimed at globally controlling and eliminating HBV. These strategies encompass universal HBV vaccination programs, prevention of mother-to-child transmission (PMTCT) interventions, mass screening programs, chronic HBV diagnosis, blood supply screening, and health education campaigns. These strategies align with recommendations from global health organizations such as the World Health Organization (WHO) and the Centers for Disease Control and Prevention (CDC), underscoring the significance of comprehensive approaches to HBV control [6,24]. The effectiveness of vaccination in reducing HBV transmission has been extensively documented [4]. Moreover, PMTCT interventions have significantly decreased the risk of perinatal HBV transmission [24]. Mass screening programs have facilitated the identification of undiagnosed cases of chronic HBV infection, enabling timely linkage to care and treatment initiation [25].

Impact of Strategies on Public Health Outcomes

The implementation of these strategies has yielded substantial improvements in public health outcomes. Numerous studies have demonstrated the effectiveness of universal HBV vaccination in reducing the incidence of new infections, preventing chronic HBV carriage, and lowering the burden of HBV-related liver diseases. Furthermore, PMTCT interventions have contributed to a reduction in perinatal HBV transmission, leading to a decrease in the incidence of chronic HBV infection among children. The introduction of universal HBV vaccination has resulted in a significant decrease in the global burden of HBV infection, particularly among children [26]. Screening programs have facilitated the early detection of HBV infection, enabling timely interventions to prevent disease progression and transmission [27].

Challenges and Gaps in HBV Elimination Efforts

Despite progress in implementing control measures, several challenges persist. These challenges encompass limited access to healthcare services, particularly in resource-constrained settings; inadequate funding for HBV programs; low awareness and stigma surrounding HBV; and disparities in vaccination coverage and healthcare access among vulnerable populations [28]. Addressing these challenges necessitates a multisectoral approach involving governments, healthcare providers, civil society organizations, international partners, and the active involvement of individuals themselves [29]. Efforts should focus on strengthening healthcare systems, improving access to HBV prevention, diagnosis, and treatment services, and raising awareness about HBV to reduce stigma and promote health-seeking behaviors [30].

Implications for Practice and Policy

The implications for practice and policy based on the findings of this study are as follows: first, there is a need to strengthen healthcare systems and enhance access to HBV prevention, diagnosis, and treatment services, particularly in underserved communities [31]. Second, targeted health education campaigns and community engagement initiatives should be implemented to raise awareness about HBV, reduce stigma, and promote health-seeking behaviors [32]. Third, policymakers should prioritize HBV elimination on national and global health agendas, allocate sufficient resources, and develop evidence-based policies to support elimination efforts [14]. Furthermore, research should focus on exploring the socioeconomic determinants of HBV transmission and developing tailored interventions to address disparities in healthcare access and outcomes [33].

Future Research Directions

Further research is warranted to fill existing knowledge gaps and improve the efficacy of HBV control and elimination strategies. Future studies could concentrate on assessing the effects of innovative interventions, such as novel diagnostic tools, antiviral therapies, and community-based interventions, on HBV outcomes [34]. Additionally, longitudinal studies are needed to monitor the long-term impact of vaccination and treatment programs on HBV incidence, morbidity, and mortality rates [35]. It is also important for research to explore the socioeconomic determinants of HBV transmission and develop tailored interventions to address disparities in healthcare access and outcomes [33,36].

## Conclusions

In conclusion, this scoping review provides a comprehensive overview of global strategies and initiatives for controlling and eliminating HBV. It acknowledges the diverse range of interventions implemented in different settings and the significant advancements made in certain regions. However, the review also recognizes the challenges that hinder HBV elimination efforts, such as limited healthcare access, financial constraints, and the persistent stigma associated with HBV.

Moving forward, it is important to address these barriers by taking concerted action, fostering collaboration among stakeholders, and prioritizing evidence-based interventions. By leveraging innovative approaches and forging partnerships, there is potential to collectively reduce the burden of HBV and improve public health outcomes on a global scale. The review emphasizes the need for further research to evaluate the effectiveness of these innovative strategies and interventions in HBV control efforts. It highlights the importance of evidence-based approaches to enhance the progress toward HBV elimination.
